# Supplementary material for: Systemic and Lower Respiratory Tract Immunity to SARS-CoV-2 Omicron and Variants in Pediatric Severe COVID-19 and Mis-C
Source: Vaccines (Basel). 2022 Feb 10;10(2):270. doi: 10.3390/vaccines10020270 (PMC8879098; doi:10.3390/vaccines10020270)
Supplement: Supplementary file 1 [file vaccines-10-00270-s001.zip › vaccines-1586964-supplementary.pdf]

Supplementary Table S1. Characteristics and neutralization titers of Plasma and EndoTracheal aspirates from pediatric COVID-19 and MIS-C patients.

Sample number	Diagnosis	Age (years)	Immuno-suppressed or given chemotherapy	SARS-CoV-2 PCR/ Antibody	Sample Type	50 % Neutralization titer								
						**WA1/2020**	**Epsilon**	**Iota**	**Kappa**	**Alpha**	**Beta**	**Gamma**	**Delta**	**Omicron**
COVID-1	COVID-19	<1	No	Positive	Plasma	132.8	117.6	83	38.2	101	34.2	57.8	112.8	10
					**EndoTracheal Aspirate**	**10**	**10**	**10**	**10**	**10**	**10**	**10**	**10**	**10**
COVID-2	COVID-19	13-18	No	Positive	Plasma	7832.4	1152.2	329.2	299.9	3705.4	143.7	324.5	1472.9	59.9
					**EndoTracheal Aspirate**	**2495**	**845**	**248**	**155**	**2433**	**89.2**	**180**	**534**	**56.4**
COVID-3	COVID-19	4-8	No	Positive	Plasma	132.2	10	10	10	59.6	10	10	10	10
					**EndoTracheal Aspirate**	**10**	**10**	**10**	**10**	**10**	**10**	**10**	**10**	**10**
COVID-4	COVID-19	13-18	No	Positive	Plasma	3755.5	3681.9	2028.3	1724.9	1797.5	214.6	505.1	3507	99.7
					**EndoTracheal Aspirate**	**45.9**	**10**	**10**	**10**	**10**	**10**	**10**	**39.5**	**10**
COVID-5	COVID-19	19-21	No	Positive	Plasma	556.6	108.3	80.3	69.5	132.2	26.5	37	94.6	10
					**EndoTracheal Aspirate**	**10**	**10**	**10**	**10**	**10**	**10**	**10**	**10**	**10**
MIS-C-1	MIS-C	9-12	No	Positive	Plasma	933	334	98.4	158	99	10	33.3	923	10
					**EndoTracheal Aspirate**	**29.8**	**10**	**10**	**10**	**10**	**10**	**10**	**28.4**	**10**
MIS-C-2	MIS-C	9-12	No	Positive	Plasma	854	70.6	44.4	35.2	53.5	10	10	48	37.8
					**EndoTracheal Aspirate**	**10**	**10**	**10**	**10**	**10**	**10**	**10**	**10**	**10**
MIS-C-3	MIS-C	4-8	No	Positive	Plasma	3249	341	89.3	116	877	27.3	45.8	458	10
					**EndoTracheal Aspirate**	**177**	**36.9**	**10**	**10**	**110**	**10**	**10**	**59.8**	**10**
MIS-C-4	MIS-C	9-12	No	Positive	Plasma	564	149	71.1	66.3	246	39	43.7	95.1	10
					**EndoTracheal Aspirate**	**21.6**	**10**	**10**	**10**	**10**	**10**	**10**	**10**	**10**
MIS-C-5	MIS-C	4-8	No	Positive	Plasma	481	10	10	10	24.7	10	10	10	10
					**EndoTracheal Aspirate**	**10**	**10**	**10**	**10**	**10**	**10**	**10**	**10**	**10**
				**Geometric mean titer**	**Plasma**	852	152	81	75	208	28	43	156	17
					**EndoTracheal Aspirate**	**32.5**	**17.8**	**13.4**	**13.2**	**22**	**12.5**	**13.4**	**22.7**	**11.9**

Supplementary Table S2: Mutations in the spike protein of SARS-CoV-2 variants used in this study.

SARS-CoV-2 variant	Mutations constructed in the spike plasmids
**Epsilon (B.1.429)**	S13I, W152C, L452R, D614G
**Iota (B.1.526)**	L5F, T95I, D253G, E484K or S477N, D614G, A701V
**Kappa (B.1.617.1)**	T95I, G142D, E154K, L452R, E484Q, D614G, P681R, Q1071H
**Alpha (B.1.1.7)**	H69-V70del, Y144del, N501Y, A570D, D614G, P681H, T716I, S982A, and D1118H
**Beta (B.1.351)**	L18F, D80A, D215G, L242-244del, R246I, K417N, E484K, N501Y, D614G, and A701V
**Gamma (P.1)**	L18F, T20N, P26S, D138Y, R190S, K417T, E484K, N501Y, H655Y, T1027I, D614G, V1176F
**Delta (B.1.617.2)**	T19R,G142D,E156del,F157del,R158G,L452R,T478K,D614G,P681R,D950N
**Omicron (B.1.1.529)**	A67V, H69-70del, T95I, G142D, V143-145del, Y145D, N211del, L212I, ins214EPE, G339D, S371L, S373P, S375F, K417N, N440K, G446S, S477N, T478K, E484A, Q493R, G496S, Q498R, N501Y, Y505H, T547K, D614G, H655Y, N679K, P681H, N764K, D796Y, N856K, Q954H, N969K, L981F

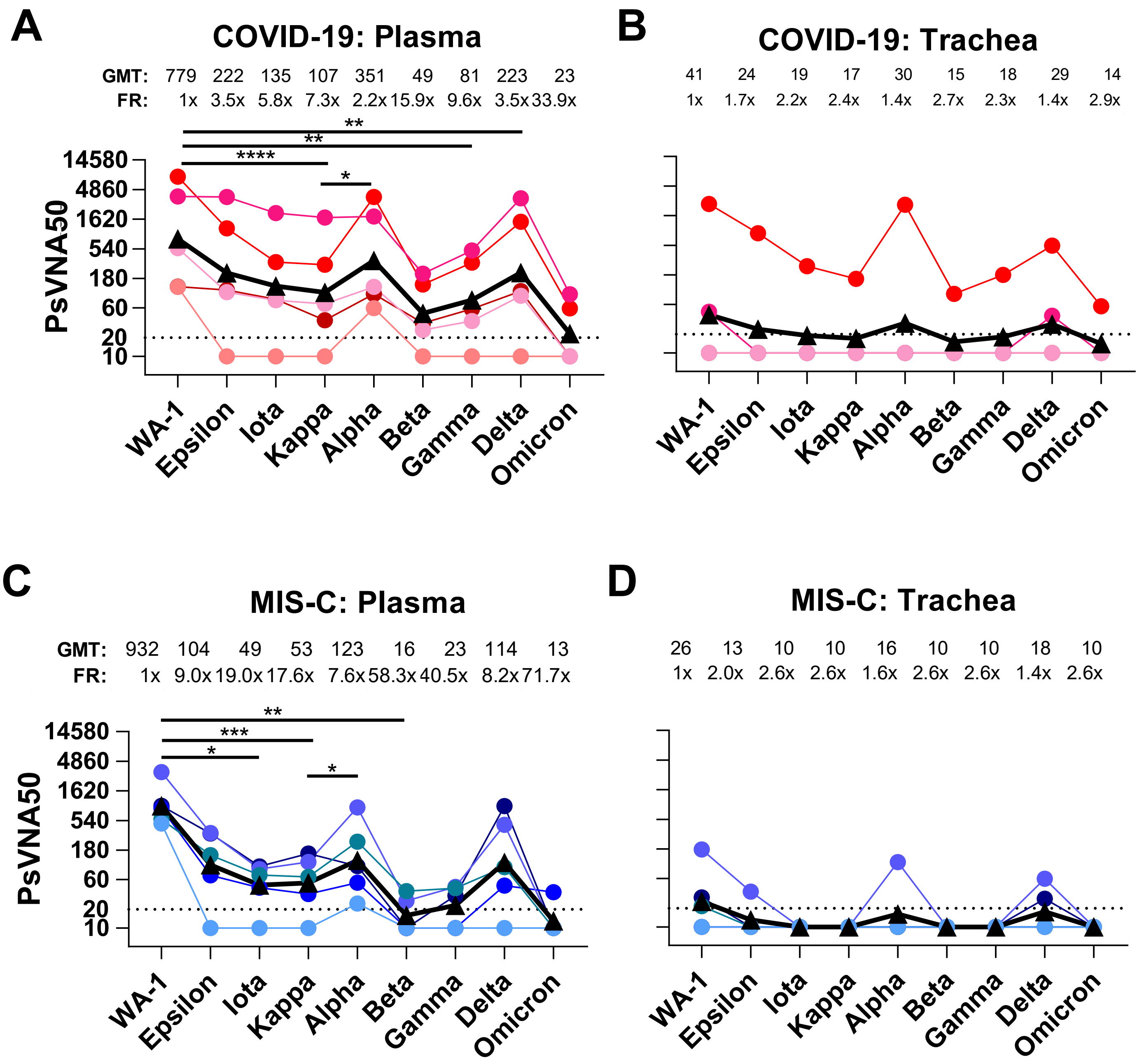

Supplementary Figure S1: Neutralizing antibody responses in plasma and endotracheal aspirates of pediatric COVID-19 and MIS-C against SARS-CoV-2 WA1/2020 strain and variant strains. Panels A-D shows SARS-CoV-2 neutralizing antibody titers of matched plasma (A & C) and endotracheal aspirates (B & D) from 5 COVID-19 (A-B) and 5 MIS-C (C-D) pediatric patients against SARS-CoV-2 WA1/2020 strain, VOIs; Epsilon, Iota, Kappa, and VOCs; Alpha, Beta, Gamma, Delta and Omicron variant by pseudovirion neutralization assay (PsVNA). PsVNA50 (50% neutralization) titer values are shown. Individual COVID-19 samples are shown with shades of red in panels in A & B, while MIS-C samples are shown with shades of blue in panels C & D. The black triangles indicate the mean PsVNA50 value against each strain. The horizontal dashed line indicates the limit of detection for the neutralization assay (PsVNA50 of 20). The numbers above the symbols indicate the PsVNA50 geometric mean titers (GMT). The geometric mean fold-reduction (FR) of SARS-CoV-2 VOIs; Epsilon, Iota, Kappa, and VOCs; Alpha, Beta, Gamma, Delta and Omicron variant neutralization titer to the ancestral WA1/2020 neutralization titer is shown. Differences between SARS-CoV-2 strains were analyzed by lme4 and emmeans packages in R using Tukey's pairwise multiple comparison test and the p-values are shown. The differences were considered statistically significant with a 95% confidence interval when the p value was less than 0.05. (*, p values of ≤0.05, **, p values of ≤0.01, ***, p values of ≤ 0.001, ****, p ≤0.0001).

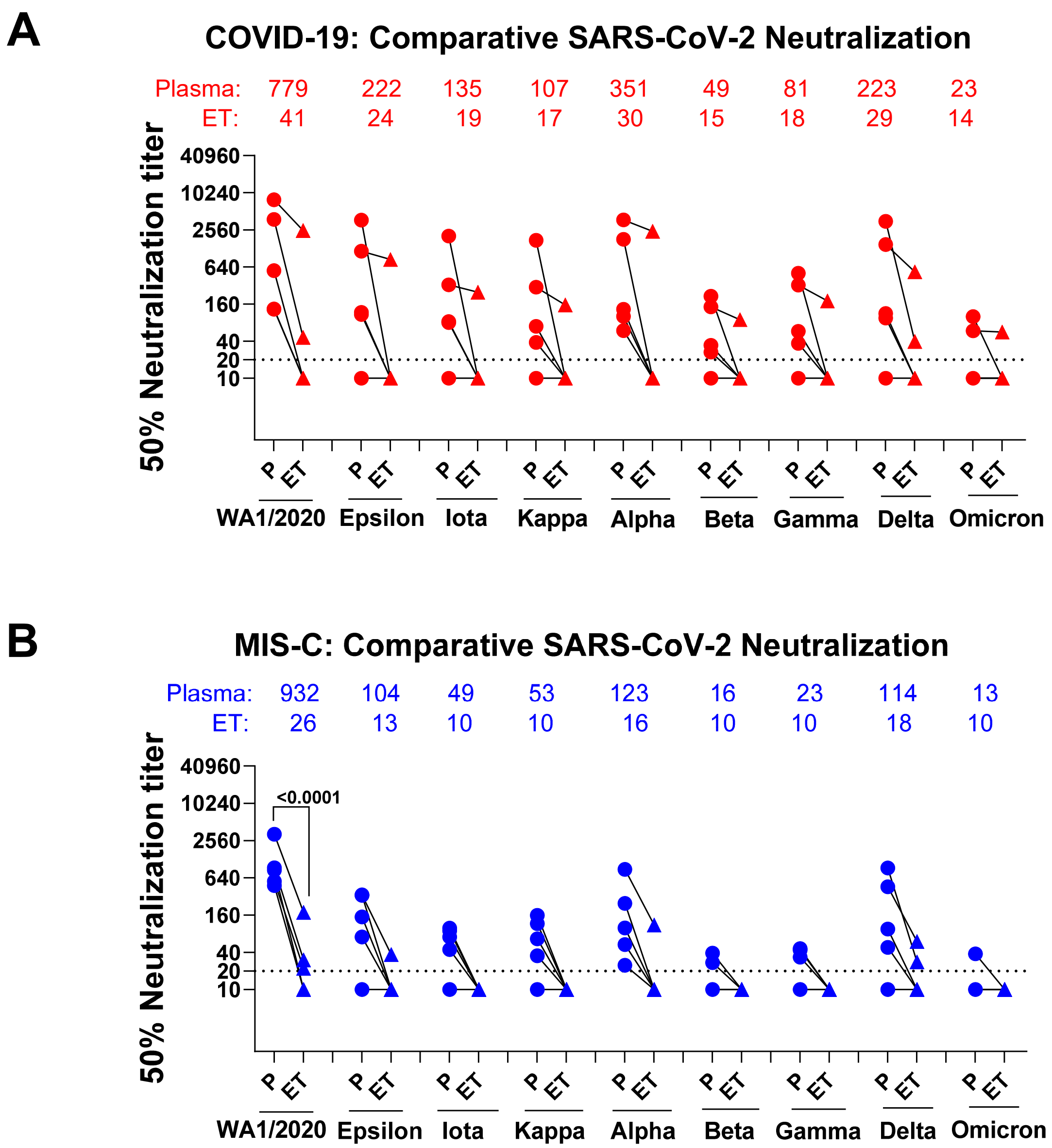

Supplementary Figure S2: Neutralizing antibody responses against SARS-CoV-2 WA1/2020 and VOCs/VOIs in matched plasma and endotracheal aspirate paired samples from pediatric COVID-19 and MIS-C patients. Panels A-B show SARS-CoV-2 neutralizing antibody titers of paired plasma (P) and endotracheal aspirates (ET) of 5 pediatric COVID-19 (panel A) and MIS-C (panel B) patients against SARS-CoV-2 WA1/2020 strain, VOIs; Epsilon, Iota, Kappa, and VOCs; Alpha, Beta, Gamma, Delta and Omicron variant as PsVNA50 (50% virus neutralization) values in pseudovirion neutralization assay (PsVNA). The horizontal dashed line indicates the limit of detection for the neutralization assay (PsVNA50 of 20). The numbers above the symbols indicate the PsVNA50 geometric mean titers (GMT) of paired plasma (P) and endotracheal aspirates (ET) against each SARS-CoV-2 strain. Differences for PsVNA50 between paired plasma (P) and endotracheal aspirates (ET) against each SARS-CoV-2 strain were analyzed by lme4 and emmeans packages in R using Tukey's pairwise multiple comparison test and the p-values are shown.
